# Sex differences in cardiovascular risk factors and quality of life among individuals with hypertension in Korea from 2013 to 2018: A cross-sectional cohort study

**DOI:** 10.1371/journal.pone.0296326

**Published:** 2024-01-02

**Authors:** Hyejin Jung

**Affiliations:** Department of Meridian & Acupoint, College of Korean Medicine, Kyung Hee University, Seoul, Republic of Korea; Navrongo Health Research Centre, GHANA

## Abstract

As its prevalence increases and its association with cardiovascular disease and mortality is established, there is a need to improve prevention and management strategies for hypertension. Therefore, we aimed to compare differences in cardiovascular health status between men and women with hypertension defined by systolic blood pressure ≥140 mmHg and diastolic blood pressure ≥90 mmHg, and/or treatment with antihypertensive drugs. The association between cardiovascular health status and health-related quality of life was also evaluated. Additionally, a secondary analysis was performed on the cross-sectional data from 7306 adults with hypertension, ≥40 years of age between 2013 and 2018. Of this sample, 51.2% of patients were women from the Korean National Health and Nutrition Examination Survey. The seven Cardiovascular Health Metrics were used to quantify cardiovascular health status and the 5-dimensional European Quality of Life Questionnaire (EQ-5D) was used to evaluate health-related quality of life. A higher proportion of men than that of women was observed in the poor cardiovascular health score group [total score 0–7], with a higher proportion of women in the intermediate group [total score 8–10] and ideal group [total 11–14] (*p*<0.0001). The proportion of women in the EQ-5D index was higher than that of men in the poor and intermediate groups (*p*<0.0001), with no difference in the proportion of men and women in the ideal group (*p* = 0.1452). Furthermore, specific cardiovascular risk factors were different between men and women: women had lower physical activity level and higher prevalence of high cholesterol (≥200 mg/dl), and men had higher body mass indices and worse diet management. These findings indicate that prevention and management strategies for hypertension and cardiovascular health might need to differ for men and women to improve outcomes.

## Introduction

The prevalence of hypertension has increased worldwide due to various risk factors including aging of the general population, unhealthy diet, and lack of physical activity [1]. Hypertension significantly increases the risk of heart, brain, kidney, and other diseases, affecting approximately 1.4 billion people worldwide [2]. Accordingly, lowering blood pressure (BP) in individuals with hypertension is an essential preventive factor for reducing cardiovascular morbidity and mortality [3–5]. In South Korea, the number of adults diagnosed with hypertension increased from 3 million in 2002 to 8.9 million in 2016 [6]. However, the awareness rate of hypertension in Korea has not increased accordingly, with a population awareness rate of 65% each in 2007 and 2016 [7]. Additionally, the treatment rate has not increased significantly, from 59% in 2007 to 61% in 2016, with hypertension control achieved in 41% of patients in 2007 and 44% in 2016, representing only a minor increase [7]. In addition, there is also evidence of lowered quality of life (QoL) in individuals with hypertension compared with that in normotensive people [8]. In general, lifestyle changes such as abstinence from alcohol and smoking, increased exercise, and dietary control are required to prevent and manage high BP, with antihypertensive medication used as needed.

Between-sex differences in the incidence, control, and management of cardiovascular disease have been reported as a result of factors such as sex hormones, genetics, and the environment [9]. Therefore, different strategies are needed to identify and manage cardiovascular health for men and women. Accordingly, our aim in this study was to evaluate differences in the cardiovascular health status of men and women with hypertension and to further investigate how overall cardiovascular health is reflected in their health-related quality of life.

## Methods and materials

### Study population and statement of ethics

A cross-sectional sample of the Korea National Health and Nutrition Examination Survey (KNHANES), conducted between 2013 and 2018 was used. The KNHANES is reviewed and approved by the Research Ethics Review Board of the Korea Centers for Disease Control and Prevention annually and is conducted in accordance with ethical standards in the 1964 Declaration of Helsinki and its later amendments. Participants provided informed consent prior to completing the KNHANES surveys, including the use of their data for research.

The KNHANES includes demographic, social, health, and nutritional information collected through health interviews, health screenings, and nutrition questionnaires [10]. The survey uses households as sampling units through a stratified and multistage cluster probability sampling design based on sex, age, and geographic area, based on household registries. To ensure an equal probability of selection, statistical weights are assigned to each participant, providing a representative sample of all non-institutionalized individuals in Korea [10].

**Fig 1:**
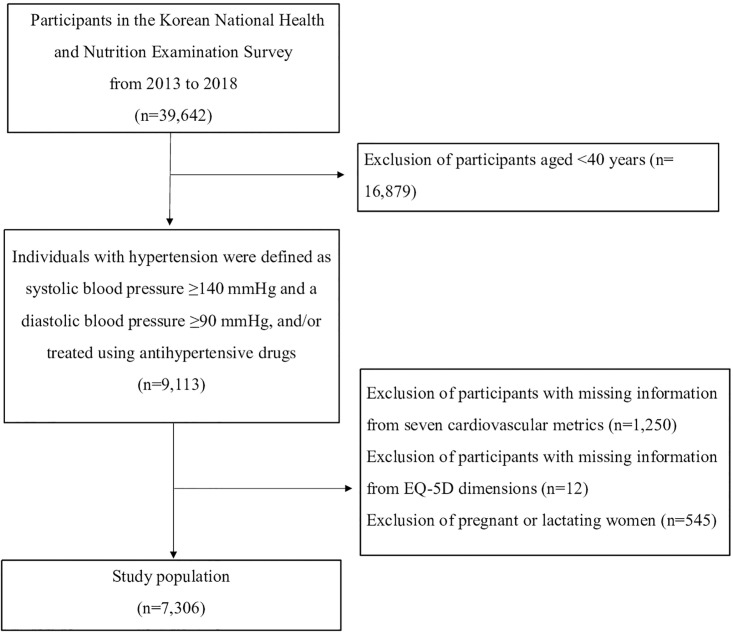
Flow chart.

In this study, we conducted a secondary analysis using online KNHANES data. Eligible data were extracted from the health surveys, checkups, and nutrition surveys completed by 39,642 individuals between 2013 and 2018. Of these data, 22,763 individuals were >40 years of age and of these, 9,113 were hypertensive with systolic BP ≥140 mmHg and diastolic BP ≥90 mmHg, and/or a history of treatment using antihypertensive drugs. After excluding individuals with missing data for the seven Cardiovascular Health Metrics and five-dimensional European Quality of Life Questionnaire (EQ-5D) as well as pregnant or lactating women, data from 7,306 were included in the analysis (Fig 1).

### Cardiovascular Health Metrics score

Cardiovascular health status was quantified using the seven Cardiovascular Health Metrics defined by the American Heart Association (AHA) [11], which include the following modifiable risk factors: smoking status, body mass index (BMI), physical activity level, diet, total cholesterol, BP, and fasting plasma glucose [12]. These risk factors were selected to measure health behaviors and biological factors associated with cardiovascular disease to inform prevention strategies [11]. Each metric was scored as 0, 1, or 2 points. Poor cardiovascular health was defined as a total score of 0 to 7, with a score of 8 to 10 indicative of intermediate health, and a score of 11 to 14 as ideal health [13]. These scores are based on the AHA guidelines, with some modifications to adapt these risk factors to a Korean population, taking race, diet, and lifestyle differences into consideration. BMI was used to classify individuals into the following three groups for analysis: 0, BMI ≥25 kg/m^2^; 1, 23–25 kg/m^2^; and 2, BMI <23 kg/m^2^ [14]. Physical activity was measured using the International Physical Activity Questionnaire (IPAQ)-short form [15], and physical activity level was classified based on the IPAQ-short form scores as follows: measured with walking, moderate-intensity activities, and vigorous-intensity activities; 0, low level; 1, moderate level; and 2, high level. Individuals were also classified into the following groups based on their dietary scores: 0, non-adherent, >2400 mg of sodium intake and low quartiles [Q1–Q3] of the Korean Healthy Eating Index [KHEI] score; 1, slightly adherent, either ≤2400 mg of sodium intake or the highest quartile [Q4] of the KHEI score; and 2, highly adherent, ≤2400 mg of sodium intake and the highest quartile [Q4] of the KHEI score [16]. The KHEI dietary evaluation index consists of 14 items. Of these, eight (breakfast, mixed grain intake, fruit intake, vegetable intake, protein foods, meat, and milk and dairy intake) are recommended for adequate consumption. Three restricted items (saturated fatty acid energy intake ratio, sodium intake, and sugar and beverage energy intake) were recommended for moderate consumption, and three (carbohydrate energy intake, fat energy intake, and total energy intake) were recommended for a balanced diet. These scores were used to evaluate the balance of energy intake. The total score of the dietary evaluation index ranged from a minimum of 0 points to a maximum of 100 points, with higher scores indicating a better diet quality [17, 18].

### Quality of life

The EQ-5D-3L dimensions include mobility, self-care, daily activities, pain/discomfort, and anxiety/depression, with responses quantified as “no problem,” “some problems,” and “severe problems.” In this study, we combined “some problems” and “severe problems” into one group titled “problems,” for analysis. The health-related QoL index taken from the EQ-5D total score was calculated using the time-trade-off method [19] as a weighted index value from 1 for the highest score and −0.171 for the lowest score.

### Other variables

To characterize the study sample, the following variables were collected: age, sex, marital status, education level, residential area, economic level, economic activity, prescription of antihypertensive drugs, and current alcohol consumption. Economic status defined by the equivalent income was calculated as the average monthly household income/number of household members and then expressed as a quartile. Alcohol intake was surveyed through self-reported responses, and the other variables were surveyed through interviews.

### Statistical analysis

Demographic data were reported as a weighted mean ± standard error (SE) or weighted percentage (%) unless otherwise indicated. Differences between men and women were evaluated using a Student’s t-test or Rao-Scott chi-squared test as appropriate for the data type and distribution, with a *p*-value <0.05 considered statistically significant. The prevalence of each Cardiovascular Health metric and each EQ-5D dimension was calculated, with differences between men and women evaluated using logistic regression and analysis of covariance (ANCOVA), adjusted for age. Differences between the poor, intermediate, and ideal groups from the five EQ-5D dimensions and total scores were evaluated according to period, age, and sex as dependent variables. The prevalence of “having a problem” was calculated for each EQ-5D dimension and the total score, with a least-squares logistic regression and ANCOVA used to evaluate differences by age and sex. As the data from the KNHANES were derived from stratified and multistage cluster probability sampling methods to represent the entire Korean population, population weighting was also applied in the analyses. The PROC SURVEY procedure was used to apply the stratification, primary sampling units, and population weights. All analyses were performed using SAS version 9.4 (SAS Institute Inc., Cary, NC, USA).

## Results and discussion

The study sample of 7,306 individuals with hypertension included 51.2% (n = 3,741) women and 48.8% (n = 3,565) men, with a mean age of 63.79 years for women and 59.30 for men (*p*<0.0001). The proportion of married individuals and those with ≥12 years of education was higher among women than among men (*p*<0.0001), while men had a higher proportion of economic activity and rate of alcohol intake than women (*p*<0.0001; Table 1).

**Table 1:** Characteristics of individuals with hypertension.

	Men	Women	*p*-value
**N**	3565	3741	
**Age (years)**	59.30 ± 0.24	63.79 ± 0.22	< .0001
**Marital status, married (%)**	95.8 (0.43)	98.7 (0.21)	< .0001
**Education level (years)**			< .0001
** ≤12**	68.1 (1.10)	88.5 (0.68)	
** >12**	31.9 (1.10)	11.5 (0.68)	
**Residential area, urban (%)**	81.5 (1.34)	81.5 (1.31)	0.9916
**Household income (%)**			0.3426
** 1Q**	24.9 (0.90)	25.4 (0.87)	
** 2Q**	23.6 (0.88)	25.0 (0.85)	
** 3Q**	26.0 (0.88)	24.1 (0.78)	
** 4Q**	25.4 (0.95)	25.4 (1.00)	
**Economic activity, yes (%)**	70.3 (0.93)	41.5 (1.06)	< .0001
**Taking antihypertensive drugs, yes (%)**	63.8 (1.04)	73.4 (0.89)	< .0001
**Current drinking, yes (%)**	31.4 (0.98)	3.9 (0.38)	< .0001

Values are reported as the weighted mean ± SE or weighted percentage (SE) unless otherwise indicated

† P-value by Student’s t-test or Rao-Scott chi-square test as appropriate

Regarding total Cardiovascular Health score, a higher proportion of women than that of men was observed in the ideal and intermediate cardiovascular health groups, with a higher proportion of men in the poor group (*p*<0.0001). With regard to individual components, a higher proportion of men than that of women who were current smokers or had quit smoking for <12 months was observed, with a higher proportion of women who were non-smokers or had quit smoking for ≥12 months (*p*<0.0001). Between-sex differences were also identified for physical activity level, with a higher proportion of women in the none group (*p* = 0.0006) and of men in the ideal group (*p* = 0.0004). The proportion of ideal BMI (<23 kg/m^2^) was higher for women than for men (*p* = 0.0027), with a greater proportion of men in the non-adherence group for diet management and a higher proportion of women in the slightly or strictly diet management group (*p*<0.0001). High total cholesterol level (≥200 mg/dl) was more prevalent in women than in men, with a higher proportion of men with low total cholesterol level (<200 mg/dl) (*p*<0.0001). Among patients with high BP, including those with BP controlled using antihypertensive drugs, when divided into ≥140/≥90 (0), 120–139/80–89 (1), and <120–80 (2) groups, no significant difference was observed between men and women. High fasting blood glucose (≥126 mg/dl and 100–125 mg/dl) was more prevalent in men than in women (*p* = 0.0026 and *p*<0.0001, respectively), with a higher prevalence for women in the ≤100 mg/dl group (*p*<0.0001).

Overall, regarding the total cardiovascular health score, calculated as the sum of all components, men had a higher prevalence of poor cardiovascular health (score 0–7) with a proportion of 60.20% (95% confidence interval (CI), 58.31–62.06) compared with a proportion of 43.81% (95% CI, 41.76–45.89) for women (*p*<0.0001). The proportion of women was higher than that of men in the intermediate cardiovascular health group (score 8–10) 49.26% (95% CI, 47.17–51.34) and 35.85% (95% CI, 34.08–37.66), respectively; *p*<0.0001) and in the ideal group (score ≥11) 6.19% (95% CI, 5.17–7.38) and 3.66% (95% CI, 3.03–4.40, respectively; *p*<0.0001; Table 2).

**Table 2:** Prevalence of Cardiovascular Health Metrics among individuals with hypertension.

	Men^a^		Women^a^		P-value
	n	Prevalence (%, 95% CI)	n	Prevalence (%, 95% CI)	
Smoking					
Current (0)	1011	28.48 (26.63–30.41)	132	3.86 (3.17–4.70)	< .0001
Quit smoking <12 months (1)	95	2.55 (2.03–3.20)	12	0.33 (0.17–0.64)	< .0001
Never or quit smoking ≥12 months (2)	2459	68.81 (66.87–70.69)	3597	95.81 (94.94–96.53)	< .0001
Physical activity					
None (0)	813	22.06 (20.40–23.81)	1120	25.92 (24.27–27.64)	0.0006
Intermediate (1)	2536	71.40 (69.54–73.20)	2486	69.73 (67.91–71.49)	0.1703
Ideal (2)	216	6.26 (5.39–7.26)	135	4.07 (3.35–4.94)	0.0004
Body mass index (kg/m^2^)					
≥25 (0)	1712	49.77 (47.85–51.70)	1758	48.17 (46.14–50.21)	0.2569
23–24.9 (1)	891	25.08 (23.41–26.84)	920	22.96 (21.37–24.62)	0.0665
<23 (2)	962	24.75 (23.20–26.38)	1063	28.48 (26.59–30.46)	0.0027
Healthy diet score					
Non-adherence (0)	1966	56.38 (54.48–58.26)	1134	31.20 (29.41–33.05)	< .0001
Slight adherence (1)	1399	38.37 (36.53–40.25)	2052	53.27 (51.27–55.26)	< .0001
High adherence (2)	200	4.82 (4.10–5.66)	555	14.19 (12.76–15.76)	< .0001
Total cholesterol (mg/dl)					
≥240 (0)	258	7.71 (6.77–8.78)	415	11.97 (10.75–13.32)	< .0001
200–239 (1)	864	24.67 (23.04–26.37)	1082	31.17 (29.42–32.97)	< .0001
<200 (2)	2443	67.51 (65.67–69.29)	2244	56.54 (54.59–58.47)	< .0001
Blood pressure (mmHg)					
≥140/≥90 (0)	1678	50.94 (48.93–52.95)	1760	50.45 (48.40–52.49)	0.7181
120–139/80–89 (1)	1207	31.35 (29.68–33.06)	1311	32.45 (30.61–34.34)	0.3717
<120–80 (2)	680	16.84 (15.43–18.35)	670	16.29 (14.87–17.82)	0.5898
Fasting blood glucose (mg/dl)					
≥126 (0)	648	17.71 (16.25–19.26)	585	14.59 (13.23–16.06)	0.0026
100–125 (1)	1533	43.24 (41.36–45.14)	1340	36.14 (34.31–38.00)	< .0001
<100 (2)	1384	38.93 (37.03–40.86)	1816	49.23 (47.35–51.11)	< .0001
Total					
Poor CV Health score (0–7)	2031	60.20 (58.31–62.06)	1584	43.81 (41.76–45.89)	< .0001
Intermediate CV Health score (8–10)	1375	35.85 (34.08–37.66)	1910	49.26 (47.17–51.34)	< .0001
Ideal CV Health score (≥11)	159	3.66 (3.03–4.40)	247	6.19 (5.17–7.38)	< .0001

^a^ Adjusted for age

P-value by logistic regression and ANCOVA

**Fig 2:**
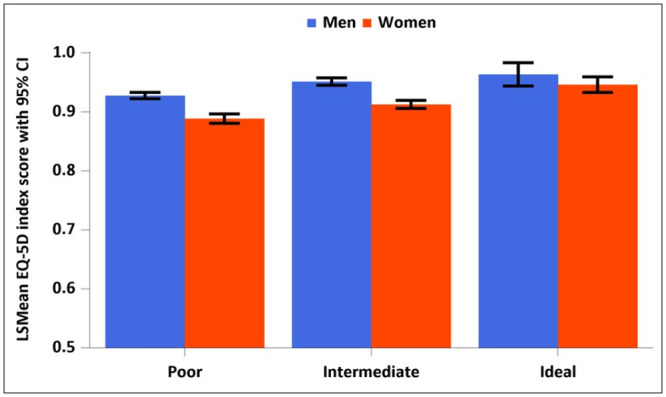
EQ-5D index score in men and women hypertensive patients. The EQ-5D index score was calculated as the least mean square after adjusting for age. *P*-values: poor group, *p*<0.0001 (men, 92.8 [95% CI 92.2–93.3]; women, 88.9 [95% CI 88.1–89.6]); intermediate group, *p*<0.0001 (men, 95.1 [95% CI 94.5–95.8]; women, 91.3 [95% CI 90.6–91.9]); ideal group, *p* = 0.1452 (men, 96.4 [95% CI 94.4–98.3]; women, 94.6 [95% CI 93.3–95.9]). EQ-5D, European Quality of Life Questionnaire.

Regarding EQ-5D index scores, in the poor and intermediate groups, women reported a higher prevalence of limitations in mobility and usual activities, pain/discomfort, and anxiety/depression than men (*p*<0.001). No between-sex differences were observed in self-care (*p* = 0.5634 and *p* = 0.129 for the poor and intermediate groups, respectively). In the ideal group, women had a higher prevalence of anxiety/depression (*p* = 0.0371), with no between-sex differences identified for the other factors (Table 3). Overall, the total EQ-5D index score was significantly lower in women than in men in the poor group (88.9 [95% CI, 88.1–89.6] and 92.8 [95% CI, 92.2–93.3], respectively; *p*<0.0001) and in the intermediate group (91.3 [95% CI, 90.6–91.9] and 95.1 [95% CI, 94.5–95.8], respectively; *p*<0.0001), with no between-sex differences in total EQ-5D in the ideal group (94.6 [95% CI, 93.3–95.9] and 96.4 [95% CI, 94.4–98.3], respectively; *p* = 0.1452; Fig 2).

**Table 3:** Distribution of health-related quality of life limitations based on the EQ-5D score.

	Men^a^	Women^a^	P
	Prevalence according to EQ-5D score (%, 95% CI)	Prevalence according to EQ-5D score (%, 95% CI)	
Poor group (0–7)			
Mobility	17.9 (15.9–20.0)	29.7 (27.0–32.5)	< .0001
Self-care	5.6 (4.5–6.9)	6.0 (4.9–7.3)	0.5634
Usual activities	9.4 (8.0–11.1)	14.5 (12.5–16.6)	< .0001
Pain/discomfort	24.0 (21.8–26.2)	36.6 (33.8–39.5)	< .0001
Anxiety/depression	9.2 (7.8–10.8)	14.8 (13.0–16.9)	< .0001
Intermediate group (8–10)			
Mobility	11.9 (10.1–14.0)	19.4 (17.3–21.6)	< .0001
Self-care	3.3 (2.4–4.5)	4.3 (3.4–5.3)	0.129
Usual activities	6.5 (5.2–8.0)	10.7 (9.3–12.4)	< .0001
Pain/discomfort	19.8 (17.3–22.5)	31.6 (29.2–34.1)	< .0001
Anxiety/depression	7.8 (6.3–9.5)	14.1 (12.5–16.0)	< .0001
Ideal group (11–14)			
Mobility	9.9 (6.3–15.2)	10.7 (7.4–15.3)	0.7807
Self-care	3.0 (1.5–6.3)	2.4 (1.2–4.8)	0.6062
Usual activities	5.8 (3.6–9.1)	5.4 (3.2–9.1)	0.8618
Pain/discomfort	16.1 (10.9–23.0)	24.3 (18.9–30.7)	0.0605
Anxiety/depression	6.0 (3.4–10.4)	12.3 (8.3–18.0)	0.0371

^a^ Adjusted for age

P-value by logistic regression and ANCOVA after the adjustment of age

We investigated differences in the prevalence of cardiovascular health indicators between men and women with hypertension in Korea for the period between 2013 and 2018. Regarding health behaviors, smoking rates declined for both men and women during this period due to Korea’s anti-smoking policy, with smoking rates remaining higher for men than for women [20]. Smoking has a greater influence on the prevalence of hypertension in men than in women, so a more sustained smoking cessation policy should be considered in men with hypertension [21].

Physical activity is a major modulating factor for the risk of cardiovascular diseases among individuals with hypertension [22]. Generally, men have higher physical activity levels than women [23]. This trend was maintained in our study sample, with a smaller proportion of men in the none group and a higher proportion in the ideal group than that of women. It is important to consider previous evidence of lower physical activity levels for individuals with than without hypertension, in both men and women [24]; therefore, active encouragement of physical activity in individuals with hypertension is important. In our study, the overall health scores were still lower in men, despite their overall higher level of physical activity. This may have contributed to the QoL in men, but additional analyses are needed.

With regards to BMI, although the proportion of women in the ideal group was greater than the proportion of men, no significant difference was observed in the proportion of men and women in the ≥23 kg/m^2^ group. A previous study reported that a BMI >25 kg/m^2^ mitigated the benefits of physical activity in men [25]. However, women undergo significant physical changes after menopause such as weight change, fat tissue redistribution, and skeletal muscle mass loss [26]. Considering these points, constant management is also necessary.

In terms of diet management, a higher proportion of women than that of men was observed in the slight and high adherence groups. Previous studies have shown lower attention to diet management by patients with than without hypertension, regardless of hypertension diagnosis [27], with diet management having a greater effect on BP reduction in men than in women [16]. Therefore, dietary management should be promoted in men with hypertension. The average level of total cholesterol tends to be higher in men than in women in early adulthood, with cholesterol levels in women rising after the age of 50 years [28], with a significant increase in the prevalence of dyslipidemia in women ≥50 [29]. In our study, we identified a significantly higher proportion of women than that of men in the ≥200 mg/dl total cholesterol level group. Therefore, cholesterol level needs to be actively managed in women with hypertension. It is generally known that men have higher fasting plasma glucose levels and a higher prevalence of diabetes than women of the same age [30]. Yet, studies have identified a greater association between high fasting blood glucose levels and coronary heart disease among women [31], with the association with stroke being higher for men [32, 33].

Health-related QoL was significantly lower in women than in men in this study, with a higher proportion of women in the poor and intermediate groups. Various factors may contribute to a lower QoL for women than for men including being older, greater difficulty for older women in receiving care due to absence of a spouse, and greater prevalence of health comorbidities [34–36]. The higher prevalence of anxiety/depression among women than among men in the ideal group may be a major factor contributing to a lower QoL among women compared with that found in men [37, 38].

Several limitations of our study need to be acknowledged. Unadjusted socioeconomic factors, the presence of comorbidities, and the severity of hypertension may have influenced the outcome. Additionally, the diagnostic criteria for hypertension are based on national survey criteria and are therefore different from the WHO criteria. The cross-sectional design prevented determination of causation for observed differences between men and women. As the data on current alcohol consumption, smoking, and physical activity were collected through self-reported responses, they may be open to bias. Despite these limitations, our study is meaningful, as it identified differences between men and women regarding the relationship between cardiovascular health status and health-related QoL among hypertensive adults in Korea.

## Conclusions

While women were shown to have better cardiovascular health status than men with a greater proportion of women in the intermediate and ideal groups, women did report a lower health-related QoL with a greater proportion of women in the poor and intermediate groups. Identified differences between men and women with regard to cardiovascular health status, QoL, and associated factors provide support for prevention and management strategies for hypertension, and associated healthcare policies that recognize differences between men and women to improve outcomes. Further research is needed to identify the underlying causes of these sex-related differences.
